# Novel Sol-Gel Synthesis of TiO_2_ Spherical Porous Nanoparticles Assemblies with Photocatalytic Activity

**DOI:** 10.3390/nano13131928

**Published:** 2023-06-25

**Authors:** Carla Calabrese, Amélie Maertens, Alessandra Piras, Carmela Aprile, Leonarda Francesca Liotta

**Affiliations:** 1 Institute for the Study of Nanostructured Materials (ISMN)-CNR, via Ugo La Malfa, 153, 90146 Palermo, Italy; carla.calabrese@ismn.cnr.it; 2 Unit of Nanomaterials Chemistry, Department of Chemistry, University of Namur, NISM, Rue de Bruxelles, 61-5000 Namur, Belgium; amelie.maertens@unamur.be (A.M.); alessandra.piras@uhasselt.be (A.P.); carmela.aprile@unamur.be (C.A.); 3 DEsign & Synthesis of INorganic materials for Energy applications (DESINe) Group, Institute for Materials Research (Imo-Imomec), Hasselt University, Agoralaan Building D, 3590 Diepenbeek, Belgium

**Keywords:** titanium, porous nanoparticles, sol-gel synthesis, anatase, rutile, photocatalysis

## Abstract

For this study, the synthesis of TiO_2_ nanomaterials was performed via a novel sol-gel method employing titanium butoxide as a metal precursor, Pluronic F127 as a templating agent, toluene as a swelling agent, and acidic water or ethanol as the reaction solvents. The method was designed by tailoring certain reaction parameters, such as the sequence of toluene addition, magnetic stirring, the type of reaction solvent, and the calcination conditions. Analysis of the specific surface area and porosity was carried out via N_2_ physisorption, whereas the morphological features of the solids were investigated via transmission electron microscopy. The crystalline structure of both the dried powders and the calcined materials was evaluated using X-ray diffraction analysis. It transpired that the different phase compositions of the solids are related to the specific synthesis medium employed. Under the adopted reaction conditions, ethanol, which was used as a reaction solvent, promoted the local arrangement of dispersed anatase particles, the specific arrangement of which does not lead to rutile transformation. Conversely, the use of water alone supported high-particle packing, evolving into a rutile phase. The photodegradation of Rhodamine B was used as a target reaction for testing the photocatalytic activity of the selected samples.

## 1. Introduction

In the broad arena of research into nanomaterials for energy and environmental and biomedical applications, titanium dioxide (TiO_2_) is one of the most widely investigated engineering materials owing to its particular features, including photocatalytic activity [1], good chemical and thermal stability, nontoxicity, and antibacterial properties [2]. Among the transition metal oxide semiconductors, TiO_2_ has emerged as significant in the areas of photocatalysis, solar cells, supercapacitors, lithium-ion batteries, gas sensors, and biosensors, among others [3,4,5,6,7,8,9,10].

TiO_2_ displays several crystalline polymorphs, which are built from specific polyhedral units. Variations between them can be ascribed to the relative distortions of the units and their different connectivity [11]. The most common crystalline phases of TiO_2_ are rutile, anatase, and brookite. The physicochemical properties of TiO_2_ are associated with certain parameters, such as the crystalline phase, size, and shape of particles. In this context, the different photoactivity of the rutile and anatase phases can be explained by the corresponding band gap energy (3.0 eV and 3.2 eV, respectively). However, such a band gap allows TiO_2_ to absorb only UV light, which covers less than 5% of the solar spectrum. In order to overcome or at least mitigate this drawback, the photoactivity of TiO_2_ can be enhanced by following two main strategies. The active spectrum of TiO_2_ can be extended through chemical or structural modifications, including the use of dopant elements, which are able to modify the TiO_2_ electronic structure. Shifting the absorption wavelength of TiO_2_ to harvest visible light as well is an emerging topic of research. In this regard, recently, Rh- and Ni-doped TiO_2_ has been used as a photocatalyst for organic synthesis along with simultaneous hydrogen production [12,13]. Elsewhere, it has been reported that nanosized TiO_2_ with a high specific surface area shows improved performance as functional building blocks for photoelectron fabrication, as well as in dye-sensitized solar cell (DSSC) applications [14]. In comparison with the TiO_2_ bulk crystalline phases, nanosized TiO_2_ particles are often made up of strained lattices due to the higher surface-to-volume ratio, leading to a greater number of available active sites. TiO_2_ nanoparticles have been prepared using different synthetic routes [15], including chemical precipitation, microemulsion, hydrothermal crystallization, and sol-gels [16]. Some researchers have reported the synthesis of TiO_2_ anatase nanocrystals, using polystyrene beads as the templating agent, allowing the control of the specific surface area (SSA) up to 172 m^2^/g for thermal treatment under N_2_. However, the surface area dramatically decreased to 65 m^2^/g when performing calcination at 450 °C under air during templating removal [17]. An approach based on the use of oil-in-water microemulsion proved useful for the preparation of mesoporous titanium oxide nanoparticles but, after calcination at 400 °C, the crystal size increased from 1 to 16 nm and the BET surface area decreased from 297 to 124 m^2^/g [18].

TiO_2_ nanoparticles (NPs) were also prepared via precipitation, using TiOSO_4_ as a precursor, and were thermally treated at 110 °C in the time range of 0–24 h. The SSA decreased from ~500 to ~255 m^2^/g by simply increasing the thermal treatment at 110 °C from 0 to 24 h. Finally, after calcination at 450 °C, the SSA was as low as 69 m^2^/g [19].

Among the abovementioned preparation procedures, the sol-gel method emerged as a bottom-up approach for synthesizing nanosized metal oxide materials that display photocatalytic activities [20,21]. However, the sintering of crystallites and the lowering of the surface area, as a result of thermal treatment, remain a major concern.

The sol-gel synthesis of TiO_2_ usually involves hydrolysis and the polycondensation reactions of titanium alkoxides, Ti(OR)_n_, allowing the formation of oxopolymers, which, in turn, give rise to an oxide network. In order to enhance the physical-chemical properties of TiO_2_ nanoparticles, some literature examples report the use of sol-gel methods adopting the triblock copolymer Pluronic F127, a non-ionic surfactant, as a structure-directing agent [22,23,24,25,26,27,28,29,30].

In the present work, we exploited novel reaction conditions in the sol-gel synthesis of nanosized TiO_2_ materials. All the materials were synthesized in the presence of Pluronic F127 as a templating agent and by using titanium butoxide as a metal precursor, toluene as a swelling agent, and acidic water or ethanol as the reaction medium. With the aim of improving the structural and textural properties of the materials, some of the reaction parameters were carefully modified. The photocatalytic activity of selected TiO_2_ solids was tested regarding the degradation of Rhodamine B under UV-A light irradiation as a proof of concept.

## 2. Materials and Methods

### 2.1. Synthesis of TiO_2_-Based Materials

All chemicals used for the synthesis were provided by Sigma Aldrich at 99.99% purity and were used without any further purification.

In order to distinguish between the dried and calcined materials, the synthesis of which is described below, the samples labeled as D were dried at 80 °C after washing and centrifugation. Samples labeled as C were calcined, usually at 400 °C, unless differently specified.

#### 2.1.1. Synthesis of **TiO_2_-H_2_O-D1**

Pluronic F127 (2.9 g) was added to HCl 1 M (aqueous solution, 320 mL) and placed in a double-jacketed glass container. The mixture was stirred at 150 rpm until the complete solubilization of the templating agent was achieved. Then, toluene (8.7 mL) was added dropwise to the templating agent solution. The resulting mixture was cooled to 4 °C without stirring for 5 h. After this time, titanium butoxide (13.3 mL, 0.379 mol) was added dropwise to the reaction mixture under stirring at 150 rpm. After 24 h of stirring at 150 rpm at 4 °C, the reaction mixture was hydrothermally treated at 80 °C for 16 h. The obtained solid was recovered by centrifugation and was then washed three times with absolute EtOH (100 mL overall). The solid was dried at 80 °C for 18 h to yield **TiO_2_-H_2_O-D1** as a white powder.

#### 2.1.2. Synthesis of **TiO_2_-EtOH-D1**

HCl 2 M (aqueous solution, 174 mL) was added to absolute ethanol (146 mL) and placed in a double-jacketed glass container. Then, Pluronic F127 (2.9 g) was added to the hydroalcoholic solution under stirring at 150 rpm until the complete solubilization of the templating agent was achieved. Then, toluene (8.7 mL) was added dropwise to the templating agent solution. The resulting mixture was cooled to 4 °C without stirring for 5 h. After this period, titanium butoxide (13.3 mL, 0.379 mol) was added dropwise to the reaction mixture under stirring at 150 rpm. After 24 h of stirring at 150 rpm at 4 °C, the reaction mixture was hydrothermally treated at 80 °C for 16 h. The obtained solid was recovered by centrifugation and washed three times with absolute EtOH (100 mL overall). The solid was dried at 80 °C for 18 h to yield **TiO_2_-EtOH-D1** as a white powder.

#### 2.1.3. Synthesis of **TiO_2_-EtOH-D2**

HCl 2 M (aqueous solution, 174 mL) was added to absolute ethanol (146 mL) and placed in a double-jacketed glass container. Then, Pluronic F127 (2.9 g) was added to the hydroalcoholic solution under stirring at 250 rpm until the complete solubilization of the templating agent was achieved. The resulting mixture was cooled to 4 °C. Then, toluene (8.7 mL) was added dropwise to the templating agent solution. After 5 h at 4 °C, without stirring, titanium butoxide (13.3 mL, 0.379 mol) was added dropwise to the reaction mixture under stirring at 250 rpm. After 1 h of stirring at 250 rpm at 4 °C, the reaction mixture was hydrothermally treated at 80 °C for 16 h. The obtained solid was recovered by centrifugation and washed three times with absolute EtOH (100 mL overall). The solid was dried at 80 °C for 18 h to yield **TiO_2_-EtOH-D2** as a white powder.

#### 2.1.4. Synthesis of **TiO_2_-H_2_O-D3**

Pluronic F127 (2.9 g) was added to HCl 1 M (aqueous solution, 320 mL) and placed in a double-jacketed glass container. The mixture was stirred at 250 rpm until the complete solubilization of the templating agent was achieved. The resulting mixture was cooled to 4 °C, without stirring, for 5 h. Then, a solution of titanium butoxide (13.3 mL, 0.379 mol) in toluene (8.7 mL) was added dropwise to the templating agent solution under stirring at 250 rpm. The reaction mixture was stirred at 250 rpm, at 4 °C, for 1 h. After this time, the reaction mixture was hydrothermally treated at 80 °C for 16 h. The obtained solid was recovered by centrifugation and washed three times with absolute EtOH (100 mL overall). The solid was dried at 80 °C for 18 h to yield **TiO_2_-H_2_O-D3** as a white powder.

#### 2.1.5. Synthesis of **TiO_2_-EtOH-D3**

HCl 2 M (aqueous solution, 174 mL) was added to absolute ethanol (146 mL) and placed in a double-jacketed glass container. Then, Pluronic F127 (2.9 g) was added to the hydroalcoholic solution under stirring at 250 rpm until the complete solubilization of the templating agent. The resulting mixture was cooled to 4 °C without stirring for 5 h. Then, a solution of titanium butoxide (13.3 mL, 0.379 mol) in toluene (8.7 mL) was added dropwise to the templating agent solution under stirring at 250 rpm. The reaction mixture was stirred at 250 rpm, at 4 °C, for 1 h. After this time, the reaction mixture was hydrothermally treated at 80 °C for 16 h. The obtained solid was recovered by centrifugation and washed three times with absolute EtOH (100 mL overall). The solid was dried at 80 °C for 18 h to yield **TiO_2_-EtOH-D3** as a white powder.

#### 2.1.6. Synthesis of **TiO_2_-H_2_O-C1, TiO_2_-EtOH-C1, TiO_2_-EtOH-C2, TiO_2_-H_2_O-C3,** and **TiO_2_-EtOH-C3**

**TiO_2_-H_2_O-D1**, **TiO_2_-EtOH-D1**, **TiO_2_-EtOH-D2, TiO_2_-H_2_O-D3,** and **TiO_2_-EtOH-D3** powders were calcined at 400 °C for 4 h, with a temperature ramp of 1.5 °C/min, to obtain **TiO_2_-H_2_O-C1**, **TiO_2_-EtOH-C1**, **TiO_2_-EtOH-C2**, **TiO_2_-H_2_O-C3,** and **TiO_2_-EtOH-C3**, respectively.

#### 2.1.7. Synthesis of **TiO_2_-EtOH-C3′**

**TiO_2_-EtOH-D3** powder was calcined at 400 °C for 5 h, with a temperature ramp of 2.0 °C/min, to obtain **TiO_2_-EtOH-C3′**.

#### 2.1.8. Synthesis of **TiO_2_-EtOH-C3″**

**TiO_2_-EtOH-D3** powder was calcined at 300 °C for 5 h, with a temperature ramp of 2.0 °C/min, to obtain **TiO_2_-EtOH-C3″**.

### 2.2. Characterization

X-ray diffraction measurement of the materials was performed on a Bruker D 5000 diffractometer equipped with a Cu Kα anode in the range of 20 to 80 (2θ), with a step size of 0.05 and a duration per step of 20 s. The crystalline phase composition of the powders was analyzed according to the ICSD database (FIZ Karlsruhe, Leibniz Institute, 2022 release, Eggenstein-Leopoldshafen, Germany). The sizes of crystallite in the titanium phases were calculated using Scherrer analyses of the most intense peaks.

Specific surface area (SSA), pore volume, and mean pore diameter of the materials were determined via N_2_ physisorption analysis at −196 °C, using an ASAP 2020 Plus instrument (Micromeritics, Norcross, GA, United States). Prior to the analysis, the samples were outgassed at 200 °C under a vacuum for 2 h. The Brunauer–Emmett–Teller (BET) method was used to calculate the SSA. The Barrett–Joyner–Halenda (BJH) method was applied to the desorption to calculate the mesoporous pore volume and the average pore diameter.

The TEM images were recorded on a Philips TECNAI 10, operating at 80 kV. Samples were deposited on a Formvar–copper grid. Particle size distribution was assessed for a minimum of 100 spherical particles.

Thermogravimetric analyses (TGA) of the dried materials were performed using a TGA/DSC1 STAR system from Mettler Toledo, Inc. Aiming to identify the proper calcination temperature of the prepared TiO_2_, the dried sample (15 mg) was subjected to (i) pre-treatment in airflow (30 mL·min^−1^) from 25 °C to 100 °C, with a heating rate of 10 °C min^−1^, then (ii) a holding time at 100 °C for 30 min, in order to remove any eventual physisorbed water or another solvent. Finally, in step (iii), the temperature was increased from 100° to 1000 °C under airflow (30 mL·min^−1^) and the weight loss that occurred during this step was taken into account. As shown in Figure S1 in the Supplementary Materials (the dried sample of TiO_2_-ETOH-D_3_ was chosen as an example), the main weight loss of around 11 wt% occurs during step (iii), between 100 and 400 °C. Therefore, unless differently specified, the samples were calcined at 400 °C, which was identified as a proper temperature by which to eliminate all the templating agent (as was also confirmed by chemical combustion analysis (CHN)) and to favor the crystallization of the titanium oxide phases (according to XRD analysis).

With the aim of determining the hydroxyl density on the surface of the calcined titanium samples, a particular procedure was used that was developed by Mrowiec-Bialoń [31]. In this procedure, a four-step TGA analysis was carried out under N_2_ flow (30 mL·min^−1^), according to the following method: (i) an isothermal step at 35 °C for 2 min; (ii) heating from 35 ° to 200 °C at a rate of 10 °C∙min^−1^; (iii) an isothermal step at 200 °C for 30 min (to remove all the physisorbed water from the open surface and inner pores); (iv) heating from 200 °C to 800 °C at a controlled rate (10 °C∙min^−1^). The hydroxyl density at the surface (as OH^−^ per nm^2^) was then computed, considering the sample weight loss in the 200–800 °C range, according to the following equation:

(1) $$\frac{(\Delta m\cdot 2N_{\mathrm{Av}}/M_{H_{2}O})}{S_{\mathrm{BET}}\cdot m_{\mathrm{sample}}}$$

where Δm is the mass loss computed in a 200–800 °C window, N_Av_ is the Avogadro number (6.022·10^23^), $M_{H_{2}O}$ is the water molecular weight (18 g·mol^−1^), S_BET_ is the specific surface area of the titanium sample, and m_sample_ is the sample mass after treatment at 200 °C, as determined by TGA.

In order to assess the residual quantity of the carbon left after calcination, chemical combustion analysis (CHN) was performed on a selected sample, labeled **TiO_2_-EtOH-C3 (calcined at 400 °C 4 h)**, using a PerkinElmer 2400 Series II analyzer. A carbon content equal to 0.19 wt % was found, confirming the effective removal of the templating agent during the washing and calcination procedures.

### 2.3. Photocatalytic Assessment

The photocatalytic performances of the selected solids were evaluated by subjecting them to UV-A light irradiation to assess the degradation of Rhodamine B (RhB), which was used as a representative dye compound. To conduct the photocatalytic experiments, we utilized a custom-made photoreactor, as previously described in Ref. [32].

### 2.4. Photodegradation in the UV-A Range

For the photodegradation tests, quartz beakers were employed, each containing 20 mL of an aqueous solution of RhB (4 mg L^−1^), along with the photocatalyst (5 mg). An axial arrangement was made with a UV-A lamp (Philips Hg lamp, 11 W, dominant wavelength 368 nm) that was positioned approximately 1 cm away from the magnetic stirrer plate among the four beakers. To maintain room temperature (24 ± 3 ℃), a fan was placed on the rear wall of the reactor. Before irradiation, the reaction mixtures were stirred in the dark for 30 min using an equal-sized magnetic stirrer bar to achieve substrate adsorption-desorption equilibrium. The reactions were stopped after 20 min. Subsequently, the suspensions were centrifuged to isolate the catalysts from the aqueous dye solution. UV-vis spectroscopy was employed to analyze the suspensions, focusing on the primary absorption peak of the dye in the visible range, specifically, at 554 nm. The percentage of degradation is determined by Equation (2):

(2) $$\mathrm{Degradation}\%=\frac{A_{0}-A}{A_{0}}\times 100$$

where A_0_ is the absorbance of the starting solution before the reaction and A is the final absorbance, measured at the end of the reaction. UV-A light irradiation was used for the photolysis tests of the RhB dye. To ensure data reproducibility, each photocatalytic test was repeated four times. The discoloration process was monitored using an Agilent Cary 5000 UV-vis spectrophotometer (Hasselt, 3500 (postcode) Belgium). The measurements were performed within the UV-visible range of 200–800 nm, employing a quartz cuvette and applying baseline corrections.

### 2.5. Recycling Tests

The reusability of the catalyst was examined in terms of the process of the photodegradation of Rhodamine B dye under UV-A light exposure. The catalyst was recovered by centrifugation, washed with water, sonicated for 15 min, and further centrifuged. Afterward, the catalyst was dried in a binding agent for 3 h at 120 °C. Once dried, it was calcined in air at 400 °C for 2 h and used for the next cycle. Conversions were estimated using Equation (2).

## 3. Results and Discussion

**Scheme 1:**
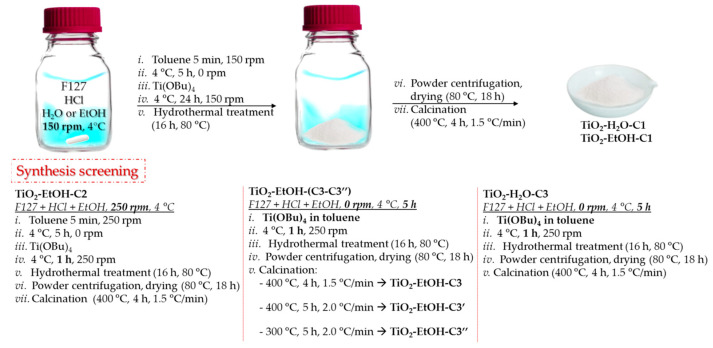
Sol-gel synthesis of TiO_2_-based materials.

The sol-gel synthesis of TiO_2_ nanomaterials was performed, using titanium butoxide as a metal precursor, Pluronic F127 as a templating agent, toluene as a swelling agent, and acidic water or ethanol as the reaction solvents. As summarized in Scheme 1, the synthetic method was designed by tailoring such reaction parameters as the sequence of toluene addition, magnetic stirring, the reaction solvent, and the calcination conditions.

The first two syntheses started with the dissolution of Pluronic F127 into acidified water or ethanol under stirring at 150 rpm. Toluene was added dropwise to the templating agent solution. This mixture was cooled to 4 °C without stirring for 5 h. After this time, titanium butoxide was added dropwise and the reaction mixture was stirred at 150 rpm. After 24 h, the synthesis mixture was hydrothermally treated at 80 °C for 16 h. The obtained solid was recovered by centrifugation, washed, and dried at 80 °C. The resulting materials, **TiO_2_-H_2_O-D1** and **TiO_2_-EtOH-D1**, were calcined at 400 °C for 4 h with a temperature ramp of 1.5 °C/min, to obtain **TiO_2_-H_2_O-C1** and **TiO_2_-EtOH-C1**, respectively.

**Figure 1:**
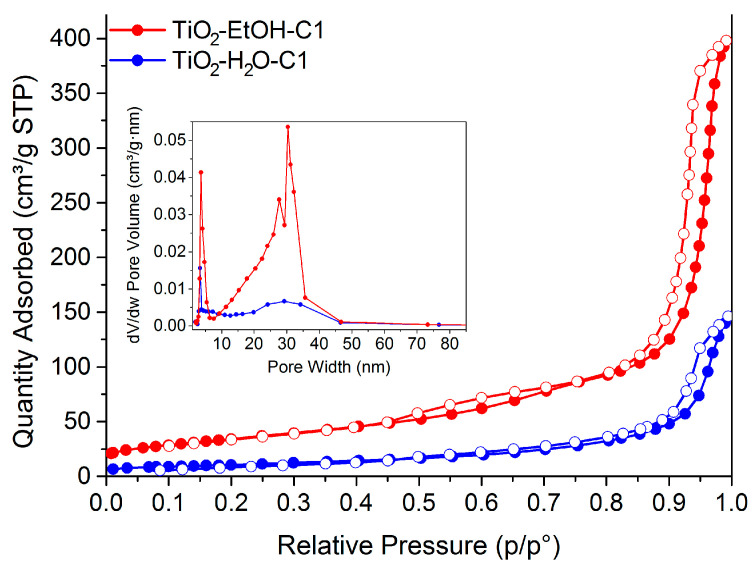
Adsorption-desorption N_2_ isotherms of **TiO_2_-EtOH-C1** and **TiO_2_-H_2_O-C1**. Inset: pore size distribution.

N_2_ physisorption analysis was performed to evaluate the textural properties of the calcined synthesized samples (Figure 1). In Table 1, the specific surface area (S_BET_) and pore volume values are listed. The N_2_ adsorption-desorption isotherm of **TiO_2_-H_2_O-C1** showed a much lower adsorbed amount of nitrogen than **TiO_2_-EtOH-C1**. In particular, **TiO_2_-H_2_O-C1** exhibited low values for specific surface area (38 m^2^/g) and pore volume (0.23 cm^3^/g). The synthesizing procedure, which was carried out by using ethanol as a reaction solvent instead of water, gave rise to a material (**TiO_2_-EtOH-C1**) with a much higher specific surface area (124 m^2^/g) and pore volume value (0.62 cm^3^/g). Moreover, the capillary condensation of the solids took place in two steps, which could be considered a combination of the filling of particle interiors at a lower p/p^0^ and the filling of the interparticle voids at a higher p/p^0^ [33]. In the inset in Figure 1, the pore size distribution of both samples is displayed.

**Figure 2:**
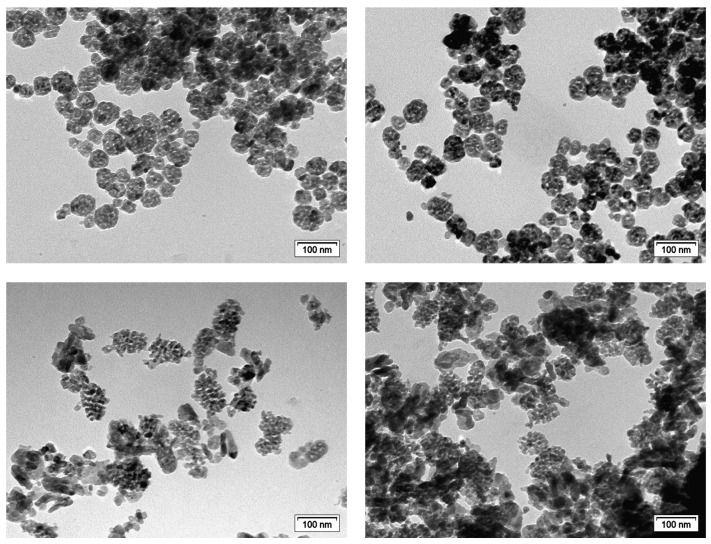
TEM micrographs of **TiO_2_-H_2_O-C1** (**top**) and **TiO_2_-EtOH-C1** (**bottom**).

The morphological features of the solids were investigated via transmission electron microscopy (TEM). TEM micrographs of **TiO_2_-H_2_O-C1** evidenced the presence of a homogeneous distribution of round-shaped nanoaggregates, with a mean particle size of 44 nm ± 11 nm over 153 particles (Figure 2). In Table 2, the particle size distribution, determined via TEM analysis of the analyzed TiO_2_-based materials, is listed.

In the case of **TiO_2_-EtOH-C1**, TEM analysis revealed a more inhomogeneous morphology made up of nanoparticles of different shapes, as evidenced in Figure 2. Due to the presence of too broad a distribution of aggregates of irregular morphology, the particle size distribution was not determined.

The crystalline structure of the calcined powders was evaluated by means of X-ray diffraction (XRD) measurements and compared with the dried ones, in order to assess if any structural modification may occur with thermal treatment.

The XRD patterns were analyzed, along with the ICSD references for anatase (n. 9854), rutile (n. 9161), and brookite (n. 31122). In Table 1, the different crystalline phases of TiO_2_-based materials, as detected by XRD, are listed.

**Figure 3:**
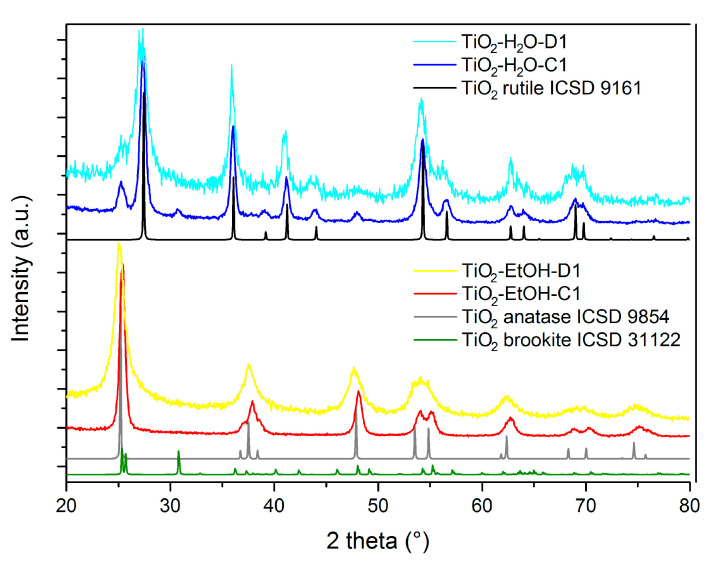
XRD patterns of reference and calcined materials.

As evidenced in Figure 3, the XRD patterns of **TiO_2_-H_2_O-D1** and **TiO_2_-H_2_O-C1** showed a crystalline structure that was characterized by the dominant presence of rutile, a reduced amount of anatase, and a very small amount of brookite. Moreover, the TiO_2_ anatase reflections were identified as the main crystalline phase in **TiO_2_-EtOH-D1** and **TiO_2_-EtOH-C1**, whereas no signals related to the rutile or brookite phases were observed. From the XRD patterns of the uncalcined solids **TiO_2_-H_2_O-D1** and **TiO_2_-EtOH-D1** to **TiO_2_-H_2_O-C1** and **TiO_2_-EtOH-C1**, it is possible to identify the formation of a more crystalline structure that is dependent on the calcination treatment.

The different phase compositions of the solids can be related to the specific synthesis conditions, which are analogous except for the use of ethanol instead of water as the reaction solvent. This observation suggests that, under the adopted reaction conditions, the use of ethanol promotes the local arrangement of more dispersed but also disordered anatase particles, the specific arrangement of which does not lead to rutile transformation, even after the calcination step. However, the sole use of water supported higher levels of particle packing, evolving into rutile phase transformation even after hydrothermal treatment at 80 °C, as evidenced by the XRD pattern of **TiO_2_-H_2_O-D1**.

The influence of the water-to-ethanol ratio on the TiO_2_ phase was reported in the literature regarding a low-temperature solvothermal route [34]. In this study, a phase compositional control over TiO_2_ was identified, which evolved from a 100% rutile phase up to a 100% anatase phase, with a progressive increase of ethanol contents. Based on the XRD-related investigations, the authors suggested that higher ethanol contents favor the anatase phase and inhibit the gain of crystallite sizes of both the anatase and rutile phases.

Moreover, it is worth mentioning that particle arrangement and packing would also impact the thermal stability and phase transformation of materials [35,36,37]. Banfield et al. [38,39] claimed that the anatase to rutile transformation could be initiated from the rutile-like elements created at the oriented contacts between anatase particles. The lack of proper particle attachment or particle coordination would decrease the possibility of rutile nucleation [40].

Starting from the synthesis conditions of **TiO_2_-EtOH-C1**, a further material, labeled **TiO_2_-EtOH-C2,** was prepared by increasing the magnetic stirring from 150 rpm up to 250 rpm. The reaction conditions of **TiO_2_-EtOH-C2** were modified in turn, with the intention of investigating if the addition procedure of toluene could influence both the textural and morphological properties of the final material. To test this hypothesis, during the synthesis of **TiO_2_-EtOH-C3**, the swelling agent and the titanium precursor were added dropwise to the surfactant solution, this being titanium butoxide that was previously dissolved in toluene. Hence, this last procedure was also performed in the absence of ethanol by using water as a reaction solvent, to yield **TiO_2_-H_2_O-C3**.

**Figure 4:**
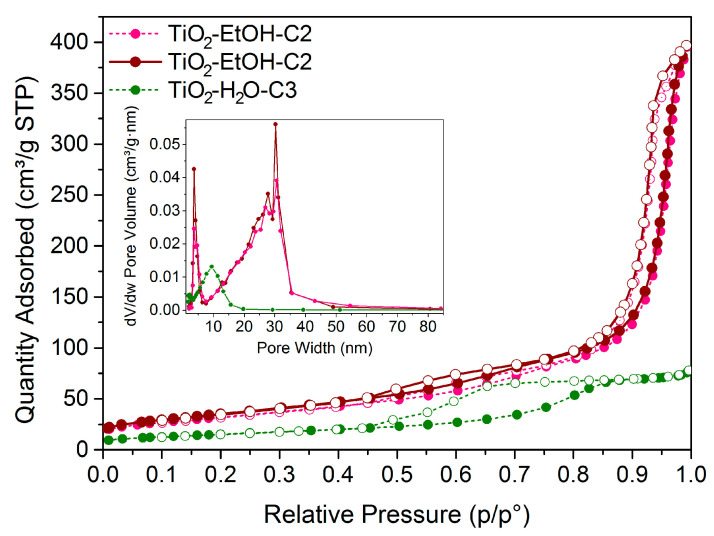
Adsorption-desorption N_2_ isotherms of **TiO_2_-EtOH-C2, TiO_2_-EtOH-C3** and **TiO_2_-H_2_O-C3**. Inset: pore size distribution.

**Figure 5:**
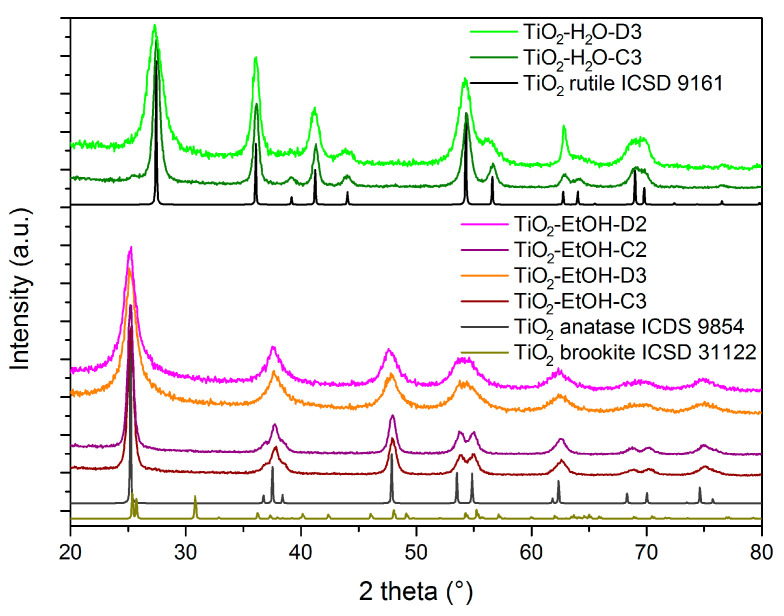
XRD patterns of the reference and calcined materials.

**Figure 6:**
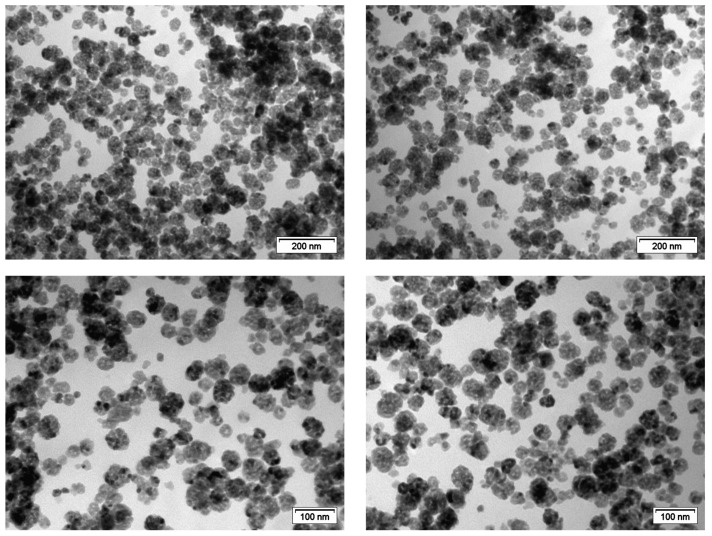
TEM micrographs of **TiO_2_-EtOH-C3**.

**Figure 7:**
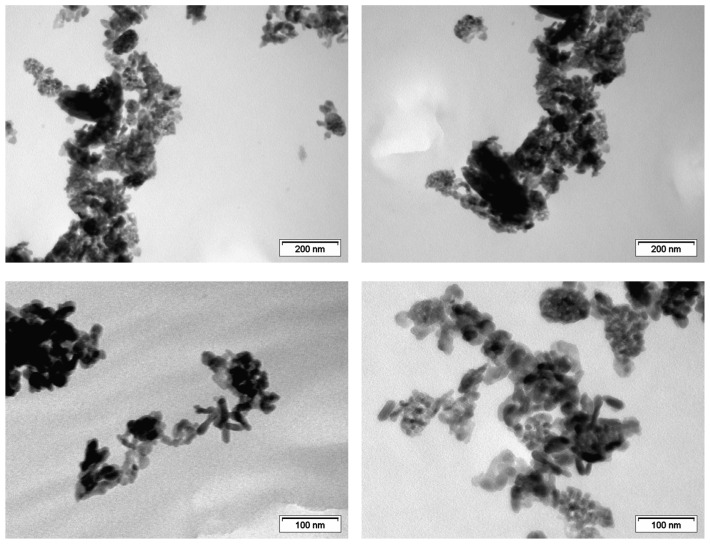
TEM micrographs of **TiO_2_-H_2_O-C3**.

All the calcined solids were characterized by means of N_2_ physisorption (Figure 4), XRD analysis (Figure 5), and TEM measurements (Figure 6 and Figure 7). In Table 1, the specific surface area (S_BET_) and pore volume values, as well as the crystalline phases of TiO_2_-based materials, are listed.

The specific surface areas of both **TiO_2_-EtOH-C2** (116 m^2^/g) and **TiO_2_-EtOH-C3** (129 m^2^/g) produced results very close to that of **TiO_2_-EtOH-C1,** whereas the pore volume value proved to be exactly the same (0.62 cm^3^/g) for all the solids prepared in ethanol. The inset in Figure 4 displays the pore size distribution for the three samples.

Moreover, the XRD patterns of **TiO_2_-EtOH-C2**, **TiO_2_-EtOH-C3,** and their dried parent materials (**TiO_2_-EtOH-D2** and **TiO_2_-EtOH-D3**) showed anatase phase peaks and fully overlapped with those of **TiO_2_-EtOH-C1** and **TiO_2_-EtOH-D1**. Conversely, TEM investigations into **TiO_2_-EtOH-C3** revealed a more ordered morphology, with the presence of homogeneous spherical assemblies of TiO_2_ nanoparticles (particle size distribution of 37 nm ± 10 nm over c.a. 100 particles).

In addition, the corresponding material was prepared in the absence of ethanol; **TiO_2_-H_2_O-C3** showed a slightly higher specific surface area (55 m^2^/g) and a lower pore volume value (0.12 cm^3^/g) than **TiO_2_-H_2_O-C1** (38 m^2^/g, 0.23 cm^3^/g) (see Table 1).

According to the XRD patterns (Figure 6), **TiO_2_-H_2_O-C3** and the corresponding dried material, **TiO_2_-H_2_O-D3**, showed a phase composition with a dominant rutile phase and a tiny amount of anatase. The crystalline structure of **TiO_2_-H_2_O-C3** was quite similar to that of **TiO_2_-H_2_O-C1**.

However, TEM micrographs of **TiO_2_-H_2_O-C3** (Figure 7) evidenced a mostly disorganized morphology, with an inhomogeneous distribution of the aggregates of TiO_2_ particles of different shapes, including rod-like and quasi-spherical objects.

**Figure 8:**
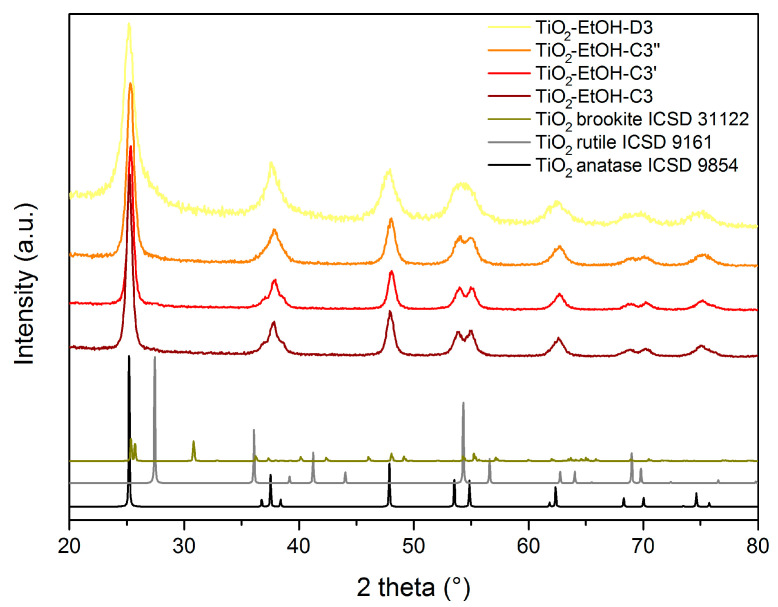
XRD patterns of the reference ICSD files, with the dried and calcined materials.

The more ordered morphology of **TiO_2_-EtOH-C3** prompted us to explore two additional calcination conditions. Specifically, two portions of the dried material **TiO_2_-EtOH-D3** were calcined as follows: (i) at 400 °C for 5 h, with a temperature ramp of 2 °C/min, to yield **TiO_2_-EtOH-C3′**; (ii) at 300 °C for 5 h, with a temperature ramp of 2 °C/min, to yield **TiO_2_-EtOH-C3**″. As previously evidenced for **TiO_2_-EtOH-C3**, the XRD patterns of both **TiO_2_-EtOH-C3′** and **TiO_2_-EtOH-C3**″ showed TiO_2_ anatase reflections as the main crystalline phase, whereas no signals of the rutile or brookite phases were detected (Figure 8). Conversely, no significant differences were observed in terms of specific surface area and pore volume values that were slightly lower than those for the sample **TiO_2_-EtOH-C3** (see Table 1).

When the calcination temperature was decreased to 300 °C (**TiO_2_-EtOH-C3**″), the XRD peaks were less resolute in comparison with the materials calcined at 400 °C (**TiO_2_-EtOH-C3** and **TiO_2_-EtOH-C3′**), while, in agreement with the lower temperature, the surface area and porosity were improved, as discussed below. Therefore, with the crystallization of the anatase phase being an important property in photocatalysis, the calcination procedure at 400 °C 4 h (1.5 °C/min) was confirmed to be the best thermal treatment.

Using the Scherrer analyses of the main anatase and rutile reflection peaks, the crystallite sizes were estimated. For all samples calcined at 400 °C, mean crystallite sizes of around 14–16 nm were found.

**Figure 9:**
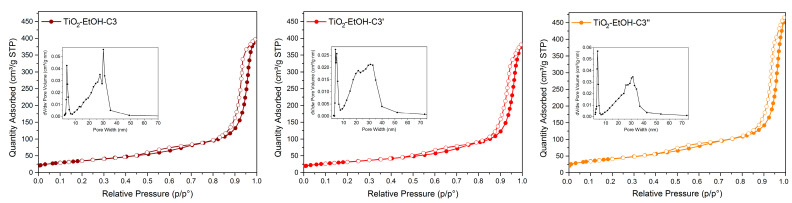
N_2_-adsorption-desorption isotherms of **TiO_2_-EtOH-C3**, **TiO_2_-EtOH-C3′**, and **TiO_2_-EtOH-C3″**. The inset displays the pore size distribution.

The N_2_ physisorption analyses showed that all the calcination treatments on **TiO_2_-EtOH-D3** allowed the obtaining of three materials with similar textural properties, as is clearly evidenced from the adsorption-desorption isotherms and pore size distributions (Figure 9). The lower calcination temperature of 300 °C gave rise to a final material with a higher specific surface area (153 m^2^/g) and a similar pore volume (0.72 cm^3^/g).

Thermogravimetric analyses were carried out over selected calcined materials (**TiO_2_-EtOH-C3, C3′** and **C3″**) under N_2_ and between 200 and 800 °C (Figure S2 in the Supplementary Materials), in order to evaluate the hydroxyl content of the surface according to a previously published procedure [31]. All samples exhibited weight losses in the range of 0.63–1.25%, corresponding to an −OH concentration of between 0.7 and 1.4 mmol g^−1^ and an OH density ranging from 3.5 to ~6.0 nm^−2^. The highest values were registered for the **TiO_2_-EtOH-C3″** sample, according to the highest surface area, and a relatively low calcination temperature of 300 °C.

Based on the reported results, it can be concluded that the synthesis of TiO_2_ samples was performed by a novel sol-gel method employing titanium butoxide as a metal precursor, Pluronic F127 for templating, toluene as a swelling agent, and ethanol as a reaction solvent, thereby producing the most orderly nanomaterial with the highest specific surface area. Such findings are in agreement with the literature. Sol-gel synthesis is based on the hydrolysis of titanium alkoxide, followed by condensation reactions, which take place, in our case, in the presence of a structural-directing agent, namely, Pluronic F127. The use of ethanol could lead to a micellar composition made up of more dispersed assemblies with a higher external surface. Ivanova et al. [41], in a study on the interaction of Pluronic F127 with water and polar water-miscible solvents, claimed that ethanol may have a preference for locating in the relatively apolar PPO-rich domains and/or for participating, together with the block copolymer, in the formation of the interface between the poly(ethylene oxide (PEO)-rich and the poly(propylene oxide (PPO)-rich domains. Hence, the increase in the interfacial area by increasing the ethanol content was related to the swelling of both the PEO and PPO blocks by the solvent.

### Catalytic Tests

Based on the characterization data, the calcined materials **TiO_2_-EtOH-C1**, **TiO_2_-EtOH-C3**, **TiO_2_-H_2_O-C1**, and **TiO_2_-H_2_O-C3** were selected for a preliminary study on their photocatalytic activity. This investigation was performed as a proof of concept to evaluate the photocatalytic performances of the solids and to give evidence of the eventual, different behaviors among the TiO_2_-based samples obtained via the different synthesis procedures. The degradation of Rhodamine B (RhB) under UV-A light irradiation was used as the target reaction. The extent (%) of RhB photodegradation is reported in Table 3. The degradation of the selected dye was followed via UV-Vis analysis of the solution after removing the heterogeneous catalyst. All the experiments were performed four times, with highly reproducible results (see Section 2.5 recycling tests section, for more details). Prior to the evaluation of the TiO_2_-based catalysts, a blank experiment was performed as well. This test indicates that a small percentage of degradation (c.a. 10%) also occurs in the absence of a catalyst as a consequence of the direct irradiation of the dye with a UV-A light source. From the data reported, the key role played by the TiO_2_ crystalline phase was clearly demonstrated. Indeed, the catalysts displaying anatase as the main phase are often more active than the solids constituted by rutile (see Table 3, where the crystalline phases of the samples were reported earlier). This behavior was previously described in the literature. Indeed, it has previously been reported that anatase has a higher charge carrier mobility and a longer lifetime than rutile, indicating that the electrons and holes generated by light can move faster and for longer before recombining. This increases the probability of its reaching the surface and initiating a reaction. Anatase also displays a lower electron affinity than rutile, thus favoring the transfer of electrons to other substances, such as oxygen or water molecules, and creating reactive oxygen species that can, in turn, oxidize organic pollutants [42,43].

The better catalytic performances of the catalysts that were prepared in the presence of EtOH can be also ascribed to a combination of favorable textural properties, such as the higher specific surface area and pore volume. Among the samples displaying the anatase phase, **TiO_2_-EtOH-C3** emerged as the most active catalyst. This behavior could be attributed to the presence of porous TiO_2_ nanoparticles, which are characterized by a homogeneous distribution of spherical shapes. The small particle size, together with the internal porosity (also visible via TEM) of the TiO_2_ domains, can favor the accessibility of RhB to the active sites, hence enhancing the catalytic activity.

**Figure 10:**
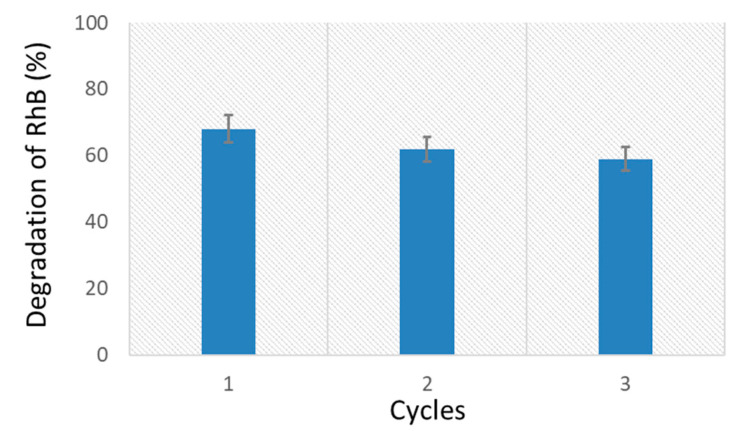
Three consecutive catalytic cycles of RhB degradation, carried out for the best solid (**TiO_2_-EtOH-C3**).

The stability of the best solid (**TiO_2_-EtOH-C3)** under the selected reaction conditions was also investigated in consecutive catalytic cycles (Figure 10). A slight decrease after the first photocatalytic cycle, followed by a stabilization of the activity, can be observed. Further investigations to better understand the recorded photocatalytic behavior and to explore the stability as a function of the different reaction conditions will be performed in the future as part of another work.

## 4. Conclusions

In this study, a novel sol-gel method was developed as an effective way to synthesize TiO_2_ nanomaterials with tunable crystalline phase compositions and morphologies. Titanium butoxide was used as a metal precursor, with Pluronic F127 as a templating agent, toluene as a swelling agent, and acidic water or ethanol as the reaction solvents. We have demonstrated that using ethanol instead of water as the reaction solvent resulted in anatase TiO_2_ nanomaterials with a higher specific surface area, pore volume, and crystallinity than those obtained when using water. Conversely, by using water as a reaction solvent in the absence of ethanol, a mostly disorganized morphology with an inhomogeneous distribution of aggregates containing particles of different shapes, including rod-like and quasi-spherical objects, was obtained.

The ordered morphology of TiO_2_ samples that were prepared in ethanol, with the formation of homogeneous spherical assemblies of nanoparticles, prompted us to explore the effect of a calcination temperature of 300 °C vs. 400 °C. It transpired that 300 °C was too low a temperature to favor the proper crystallization of the anatase phase, which proved to be the key phase in photocatalysis.

Selected TiO_2_ samples were investigated regarding the degradation of the Rhodamine B (RhB) under UV-A light irradiation, with some of them exhibiting promising photocatalytic activity. The anatase phase materials prepared in ethanol showed better photocatalytic performance than those prepared in water; thus, these are good candidates for environmental applications.

## Figures and Tables

**Table 1:** Specific surface area (S_BET_), pore volume, and crystalline phases of TiO_2_ calcined materials.

Material	Calcination Conditions	S_BET_ (m^2^/g)	Pore Volume (BJH_Ads._) (cm^3^/g)	Main Crystalline Phase
**TiO_2_-H_2_O-C1**	400 °C, 1.5 °C/min, 4 h	38	0.23	Rutile
**TiO_2_-H_2_O-C3**	400 °C, 1.5 °C/min, 4 h	55	0.12	Rutile
**TiO_2_-EtOH-C1**	400 °C, 1.5 °C/min, 4 h	124	0.62	Anatase
**TiO_2_-EtOH-C2**	400 °C, 1.5 °C/min, 4 h	116	0.62	Anatase
**TiO_2_-EtOH-C3**	400 °C, 1.5 °C/min, 4 h	129	0.63	Anatase
**TiO_2_-EtOH-C3’**	400 °C, 2 °C/min, 5 h	116	0.59	Anatase
**TiO_2_-EtOH-C3’’**	300 °C, 2 °C/min, 5 h	153	0.72	Anatase

**Table 2:** Particle size distribution of selected samples, as determined by TEM analysis over a minimum of 100 particles.

Material	Particle Size Distribution (nm) via TEM Analysis
**TiO_2_-H_2_O-C1**	44 nm ± 11 nm
**TiO_2_-H_2_O-C3**	n.d.*
**TiO_2_-EtOH-C1**	n.d.*
**TiO_2_-EtOH-C3**	37 nm ± 10 nm

n.d.*: The particle size distribution was not determined for the samples that displayed too broad a distribution of aggregates of irregular morphology.

**Table 3:** Extent (%) of RhB photodegradation over selected titanium samples, carried out under UV-A light irradiation.

Material	Main TiO_2_ Crystalline Phase	Photodegradation RhB (%)
**TiO_2_-H_2_O-C1**	Rutile	25 ± 4
**TiO_2_-H_2_O-C3**	Rutile	15 ± 1
**TiO_2_-EtOH-C1**	Anatase	37 ± 3
**TiO_2_-EtOH-C3**	Anatase	68 ± 5

## Data Availability

The data presented in this study are available in the article.

## Supplementary Materials

The following supporting information can be downloaded at: https://www.mdpi.com/article/10.3390/nano13131928/s1, Figure S1: TGA profile of a selected dried sample (TiO_2_-ETOH-D3 under air flow (step (iii), see experimental part) with the aim to investigate the thermal decomposition of the templating agent; Figure S2: TGA profiles of selected titania samples for determining the hydroxyl content: step (iv)) heating from 200 °C to 800 °C at controlled rate (10 °C∙min-1) under N_2_ flow.
