# Supplementary material for: Novel Sol-Gel Synthesis of TiO2 Spherical Porous Nanoparticles Assemblies with Photocatalytic Activity
Source: Nanomaterials (Basel). 2023 Jun 25;13(13):1928. doi: 10.3390/nano13131928 (PMC10343489; doi:10.3390/nano13131928)
Supplement: Supplementary file 1 [file nanomaterials-13-01928-s001.zip › nanomaterials-2422130-supplementary.pdf]

Supporting information

Article

Novel Sol-Gel Synthesis of TiO_2 Spherical Porous Nanoparticles Assemblies with Photocatalytic Activity

Carla Calabrese [1], Amélie Maertens [2], Alessandra Piras [2,3], Carmela Aprile [2] and Leonarda Francesca Liotta [1,*]

[1] Institute for the Study of Nanostructured Materials (ISMN)-CNR, via Ugo La Malfa, 153, 90146 Palermo, Italy; carla.calabrese@ismn.cnr.it
[2] Unit of Nanomaterials Chemistry, Department of Chemistry, University of Namur, NISM, Rue de Bruxelles, 61-5000 Namur, Belgium; amelie.maertens@unamur.be (A.M.); carmela.aprile@unamur.be (C.A.)
[3] DEsign & Synthesis of INorganic materials for Energy applications (DESINe) Group, Institute for Materials Research (Imo-Imomec), Hasselt University, Agoralaan Building D, 3590 Diepenbeek, Belgium; alessandra.piras@uhasselt.be (A.P.).
* Correspondence: leonardafrancesca.liotta@cnr.it

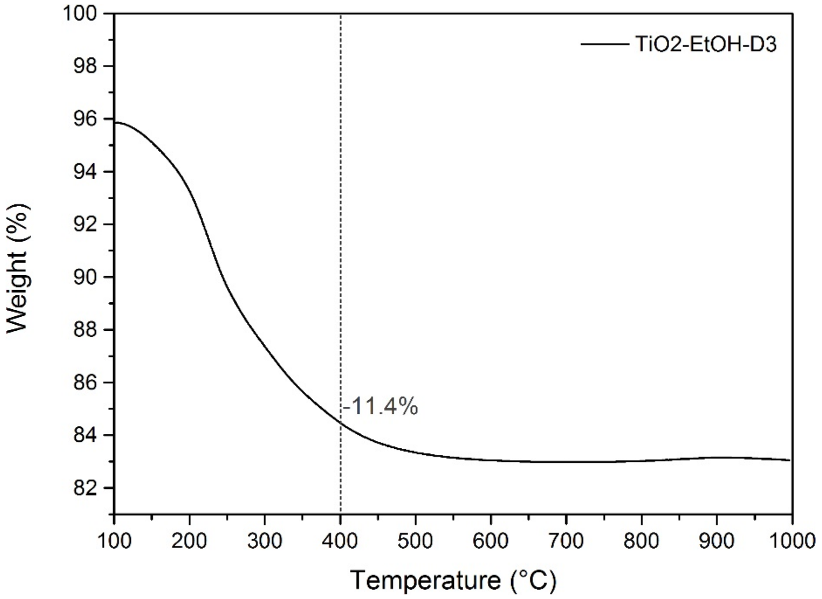

Figure S1. TGA profile of a selected **dried sample** (TiO_2-ETOH-D_3 under air flow (step (iii), see experimental part) with the aim to investigate the thermal decomposition of the templating agent.

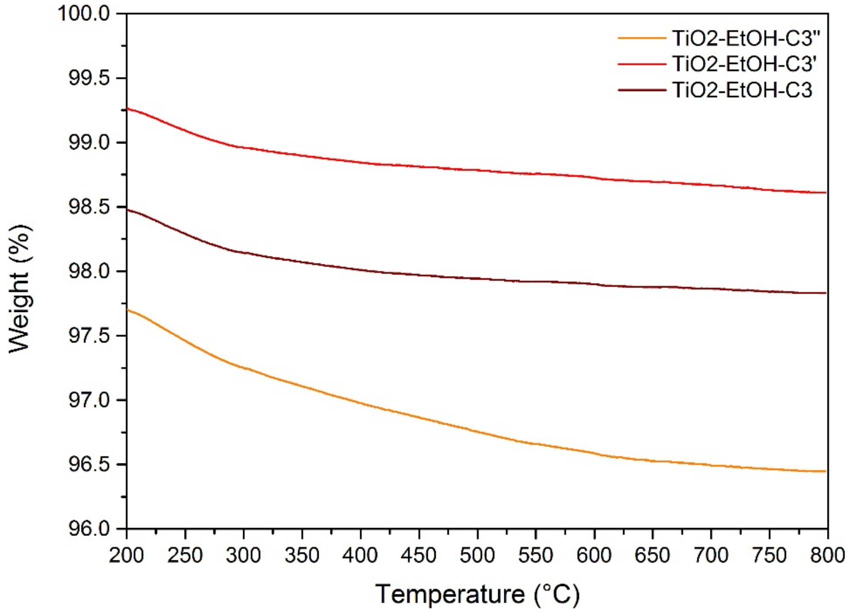

Figure S2. TGA profiles of selected titania samples for determining the hydroxyl content**:** step iv) heating from 200 °C to 800 °C at controlled rate (10 °C·min^{-1}) under N_2 flow (see experimental part).
